# A Review of Treatments for Iliotibial Band Syndrome in the Athletic Population

**DOI:** 10.1155/2013/367169

**Published:** 2013-10-02

**Authors:** Corey Beals, David Flanigan

**Affiliations:** ^1^Department of Surgery, Wexner Medical Center, 395 W. 12th Avenue Columbus, OH 43210, USA; ^2^Department of Sports Medicine, Wexner Medical Center, 2050 Kenny Road, Suite 3100 Columbus, OH 43221, USA

## Abstract

Iliotibial band syndrome (ITBS) is a common injury in runners and other long distance athletes with the best management options not clearly established. This review outlines both the conservative and surgical options for the treatment of iliotibial band syndrome in the athletic population. Ten studies met the inclusion criteria by focusing on the athletic population in their discussion of the treatment for iliotibial band syndrome, both conservative and surgical. Conservative management consisting of a combination of rest (2–6 weeks), stretching, pain management, and modification of running habits produced a 44% complete cure rate, with return to sport at 8 weeks and a 91.7% cure rate with return to sport at 6 months after injury. Surgical therapy, often only used for refractory cases, consisted of excision or release of the pathologic distal portion of the iliotibial band or bursectomy. Those studies focusing on the excision or release of the pathologic distal portion of the iliotibial band showed a 100% return to sport rate at both 7 weeks and 3 months after injury. Despite many options for both surgical and conservative treatment, there has yet to be consensus on one standard of care. Certain treatments, both conservative and surgical, in our review are shown to be more effective than others; however, further research is needed to delineate the true pathophysiology of iliotibial band syndrome in athletes, as well as the optimal treatment regimen.

## 1. Introduction

Iliotibial band syndrome is a common knee injury caused by inflammation of the distal portion of the iliotibial band (ITB), which results in lateral knee pain. The distal iliotibial band slides over the lateral femoral epicondyle, and during repetitive flexion and extension activities of the knee excessive friction and potential irritation results in pain. Potential risk factors for the development of iliotibial band syndrome include preexisting iliotibial band tightness, high weekly mileage, time spent walking or running on a track, interval training, and muscular weakness of knee extensors, flexors, and hip abductors [1, 2]. Populations who expose their knees to a greater amount of flexion and extension activities, such as athletes, particularly long distance athletes, put themselves at a higher risk for iliotibial band syndrome. Due to the pathophysiology of IT band syndrome, runners have been a group often looked at for prevalence and management of this syndrome. ITB syndrome has been documented to have as high as a 22.2% incidence of all lower extremity injuries in runners [3]. Despite a clear pathophysiology, it is unclear why this syndrome affects some athletes greater than others. Few studies have shown any direct relationship between biomechanical factors and the development of iliotibial bad syndrome [1, 2, 4–6]. 

Athletes with ITB syndrome typically complain of a sharp or burning pain roughly 2 cm superior to the lateral joint line [3]. The pain may radiate proximally or distally, and in less severe cases, the pain may quickly subside upon cessation of activities. Often pain will occur as activities proceed. It is not uncommon that the athlete will experience popping on the lateral aspect of the knee with activities. 

ITB syndrome is a clinical diagnosis and most often additional diagnostic studies are not necessary. It should be suspected in overuse and nontraumatic cases of knee pain where rest has not been helpful. Ober's test is one of numerous physical exam tests often used to assess the tightness of the ITB. If the leg can be passively stretched to a position horizontal but not completely adducted to a table, this constitutes minimal tightness. If the leg can be passively adducted to horizontal at best, this constitutes moderate tightness, and if it cannot be passively adducted to horizontal, this is maximal tightness [7]. Popping of the ITB over the lateral femoral condyle can also occur in this position as the knee is brought through range of motion. Palpation over the ITB during this maneuver typically will reproduce pain. History, however, is much more important than physical exam in diagnosis and short-term resolution of symptoms following corticosteroid injection and be both diagnostic and therapeutic.

MRI may be of use if there is doubt about the diagnosis as well as to exclude an intra-articular problem such as a lateral meniscal tear; however, isolated ITBFS often does not lead to MRI abnormalities and can be misdiagnosed if a minor but different lesion is present. Two studies revealed that in patients with iliotibial band syndrome, MRI studies have shown that the distal portion of the ITB may thicken, and a bursa deep to the iliotibial band over the lateral epicondyle becomes inflamed and filled with fluid [8, 9]. When the athletic population was isolated, normal, or cystic, poorly defined signal intensities at the distal portion of the ITB predominated. Only in chronic cases was a thickening of the distal ITB at the level of the lateral femoral epicondyle seen [8].

While the majority of patients respond to a nonsurgical, conservative approach, this does not occur for all, and escalation of treatment is necessary. This is especially true in athletes that present with refractory cases, and at this time surgical intervention can be used [3]. Unfortunately, refractory cases can occur quite often, and no treatment has been shown to work best. The purpose of this review is to outline both the conservative and surgical options for treatment of iliotibial band syndrome in athletes.

## 2. Methods

**Figure 1:**
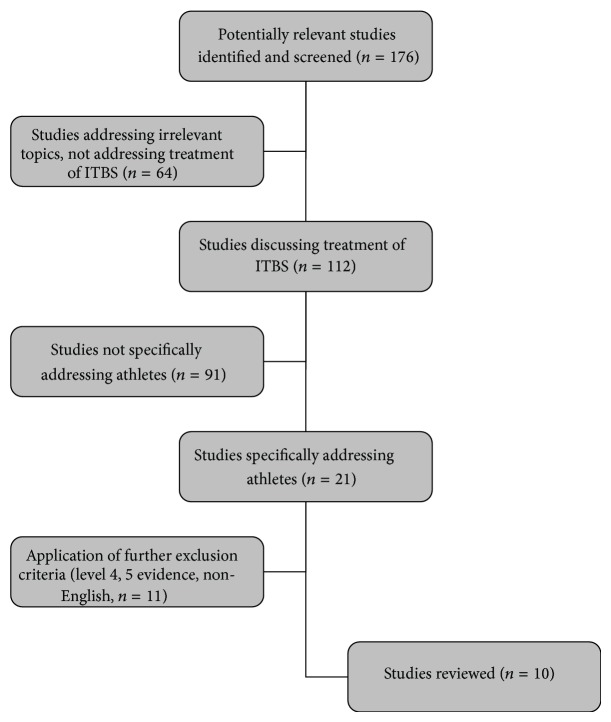
Flow chart displaying search process for review.

In order to find the most current treatment options for ITB syndrome in athletes, a literature search was conducted in the PubMed database. Criteria for inclusion in this review were papers that primarily (but not exclusively) focused on the athletic population, achieved a level III or greater level of evidence, addressed therapeutic options for ITB syndrome (conservative or surgical), and were written in English. An initial search of iliotibial band syndrome yielded 176 results. After limiting the results to those articles that discussed treatment options, and focused on the athletic population, 10 articles were reviewed.  Figure 1 outlines the sequence of the literature search. 

## 3. Results

### 3.1. Conservative Treatment

There are many different conservative treatment modalities for IT band syndrome. Many of these treatment modalities have been geared toward the runner population, and certain guidelines to return to sport (running) have been suggested [3].  Table 1 illustrates four studies that outlined conservative treatment modalities.

In a randomized controlled trial (RCT), Schwellnus et al. investigated the effect of initial treatment (day 0–7: rest, ice application, and medication) in 43 patients with unilateral ITBS. All subjects received physical therapy consisting of ultrasound, deep transverse friction massages on days 3, 5, and 7 and daily stretching of the IT band. Medication was delivered over the 7 days in a double-blind, placebo-controlled fashion with group 1 taking a placebo anti-inflammatory, group 2 an anti-inflammatory (Voltaren), and group 3 an anti-inflammatory/analgesic (Myprodol). Compared with the other groups, group 3 had less pain during running from day 3 onward, and their running time/distance on the treadmill significantly increased from day 0 to 7 [10].

In another randomized controlled trial, 18 runners with acuteonset ITB syndrome (<14 days' duration) were randomly assigned into two groups: group 1 received a corticosteroid injection and group 2 received a placebo injection. Subjects were instructed not to run for 14 days following the injection and to apply ice to the area for 30 minutes every 12 hours. Running pain was significantly decreased in the group that received the corticosteroid injection [11]. 

In a case series of 196 running injuries, Pinshaw et al. found ITBS to the third most common injury (12%) behind peripatellar pain syndrome (22%) and posterior tibial stress syndrome (18%). In those with ITBS a four-step conservative approach was used for treatment. These steps included running shoes: change to softer shoes, use of in-shoe support and shoe alterations, and/or removal of the outside heel flare of the shoe for the injured side. Leg-length discrepancies: adapt shoe of the shorter leg by adding material to the mid-sole to ensure 100% correction at the heel, 50% correction in the mid-sole, and 25% correction at the ball of the foot. Training methods: if appropriate, reduce training distance and decrease running speed and amount of hill running. Also, incorporate a sufficient number of days for recovery. Ice application: apply ice to the injured area for 30 minutes twice a day [12].

Finally, in a case series done at the Stanford Sports Medicine Clinic, 24 runners (10 M, 14 W) with ITBS completed a 6-week rehabilitation program, which consisted of local application of ultrasound with corticosteroid gel for the first two sessions. All patients were instructed to stretch the IT band three times a day, and hip abduction exercises and pelvic drop sets to strengthen the gluteus were increased throughout the program with a goal of 3 sets of 30 repetitions. Nonsteroidal anti-inflammatory drugs were prescribed until the patients were free of pain during daily activities. The investigators found a mean increase of 34.9% and 51.4% in the injured leg of the hip abductor torque for females and males, respectively. Twenty-two of the 24 athletes (91.7%) of the athletes were able to return to running at the end of the 6-week program [2].

These four studies demonstrate how diverse conservative treatment for ITBS can be. From clinical experience, rest is the best treatment for the acute cases. This treatment becomes less useful as it becomes a more chronic condition when bursal and periosteal changes have set in. There is limited evidence to support one specific approach to the treatment of ITBS; however, when looking at the desired goal of return to sport, a combination of rest (2–6 weeks), stretching, pain management, and modification of running habits produces a high return to sport rate. 

### 3.2. Surgical Treatment

Surgery is often reserved for refractory cases that have failed other avenues of conservative management. However, in the athletic population, return to sport is a common concern, and multiple, long absences from sport due to trials of various conservative treatment approaches are often not ideal. 

There are differing viewpoints as to when surgical treatment should be implemented. Martens et al. suggests that conservative treatments should be maintained for an average of 9 months before consideration of surgical intervention [13]. Others have based their decision for surgical intervention on the observation that at 30 degrees flexion, the posterior fibers of the ITB are tighter against the lateral femoral epicondyle than are the more anterior fibers, in which case a surgical release in the posterior fibers is needed to correct the problem [14, 15].

In one study, 36 athletes with a resistant ITBS were treated with a standard arthroscopic technique, limited to the resection of lateral synovial recess. The patients had suffered from ITBS for an average of 18 months (1–7 years). Thirty-three patients (mean age 31.1 years) were available for followup at least 6 months postoperatively. Prior to surgery, all patients had been treated conservatively for at least 6 months with rest, correction of training error, shoe modification, physical therapy and local infiltration with steroids. Thirty-two patients had good or excellent results based on subjective functional results at followup. All patients went back to sports after 3 months. In 2 patients a meniscal lesion was found, which required treatment, and an associated cartilage lesion of the femoral condyle was found in the one patient that reported a fair outcome at followup. The author concluded that arthroscopic resection of the lateral synovial recess in resistant ITBS is a valid option with a consistently good outcome, which also allows excluding or treating other intra-articular pathology [16].

A retrospective study on athletes in Norway looked at 45 patients who failed conservative management of ITBS. The surgical procedure of choice for these resistant ITBS cases was transection of the posterior half of the iliotibial band where it passes over the lateral epicondyle of the femur. With a mean age of 27 (14–46) years, 38 (84.4%) had excellent or good results, 6 (13.3%) had fair results, and 1 (2.3%) had a poor result [17]. Return to sport was not documented in this study, but 75.6% of patients reported that they would have the operation again [17].

Bursectomy has also been explored as a surgical treatment option for ITBS. In a recent study a single surgeon performed 11 open iliotibial band bursectomies on 11 patients (7 M, 4 W). Each patient presented with persistent (>6 months) symptoms despite conservative treatment, with an average age at onset of 29 (24–41) years [18]. After a minimum of 20-month followup, all patients were able to return to their preinjury Tegner activity levels, and all reported less pain (11-point visual analogue scale score decreased by 6 points) [18]. Nine of the 11 patients said that knowing what they know now they would have the surgery performed again for the same problem. This population, however, was a mix of athletic and the general population, and the study did not separate out the results of each population. 

## 4. Discussion

Iliotibial band syndrome (also called iliotibial band friction syndrome) is a common problem encountered in the knees' of athletes, especially endurance athletes whose sport requires repetitive knee flexion. ITBS can often recur in the athletic population, causing significant morbidity and delay in return to sport [19]. There is debate on whether iliotibial band syndrome is truly a friction syndrome where the ITB itself is pathologic or whether a pathologic bursa forms between the ITB and the lateral femoral condyle, causing the pain. This is an important concept because successful surgical treatment of the syndrome must address the underlying pathological causes [18].

Regardless of stance on the pathophysiology, conservative management is the first line of therapy for ITBS. However, both conservative and surgical therapies play a major role in recalcitrant cases. A combination of therapies (rest, pain relief, stretching, strength training, and running habit modification) works best for returning athletes to their preinjury level and reducing their symptoms. However, a systematic regimen involving all aspects of conservative therapy has not been established. A recent systematic review on iliotibial band syndrome in runners concluded that there is limited evidence to support one specific approach to the diagnosis and treatment of ITBS, suggesting that additional research is needed to elucidate an optimal treatment regimen [20]. In our review, conservative therapy alone was found to have a 44% complete cure rate with return to sport at 8 weeks and a 91.7% return to sport rate at 6 months [2, 12].

ITB syndrome pathophysiology plays a key role in guiding surgical treatments. Most surgeons who have published in the literature ascribe to the iliotibial friction band syndrome theory, and numerous procedures that excise or release this supposedly pathologic portion of the ITB have been described. Cortisone injections should still be used first in these scenarios, as ITBS is considered an inflammatory friction syndrome. However, as the duration of symptoms increases and conservative measures fail, surgical treatment may be needed for resolution of symptoms. Three of these procedures have been described above, all in athletes, with a return to sport rate of 100% at an average of 3 months [16] and 7 weeks [13]. One study did not report return to sport rate [17]. Furthermore, Fairclough et al. did not find a bursa in either the 6 cadaveric specimens they dissected or the 2 symptomatic patients on whom they performed an MRI [21]. In patients with iliotibial band syndrome MRI findings of the ITB can be normal. In a study of 16 patients with ITBS, 31% had a discrete fluid collection medial to the ITB, with a normal looking ITB. However, it was hypothesized that this collection likely arose from chronic inflammation beneath the ITB, resulting in the formation of a secondary or adventitious bursa rather than from the inflammation of an existing primary bursa [22].

There are also those that ascribe to the theory that the ITB itself is not pathological in patients with iliotibial band syndrome, but rather the pain and functional deficits are generated by a pathological bursa that forms beneath the ITB due to compression of that underlying tissue rather than a friction mechanism. In the study performed by Hariri et al. described above, they consistently found what appears to be an inflamed bursa underlying a benign-appearing ITB [18]. This study, however, was not done solely on the athletic population whereas the ones described which favor the friction theory of iliotibial band syndrome were. Surgical intervention is often only utilized after patients have failed conservative management for ITBS, making return to preinjury level a difficult task. Although there are two theories on the pathophysiology of ITBS, when looking at return to sport rate in the athletic population, resection of the lateral synovial recess, after failure of conservative therapy provides an excellent return to sport rate.

## 5. Conclusion

Iliotibial band syndrome is a common cause of lateral knee pain in the athlete, especially runners and other endurance athletes [23]. Both conservative and surgical approaches are viable treatment options, and both need to be considered during treatment planning. While the majority of cases resolve with conservative management, resistant cases are seen in many athletes, requiring surgical intervention. The cases that require surgical intervention are often chronic in nature, and it is important to recognize the duration of symptoms so that surgical treatment can be initiated early. Despite many options for both surgical and conservative treatment, there has yet to be consensus on one standard of care. Further research is needed to delineate the true pathology behind iliotibial band syndrome in athletes, as well as the optimal treatment regimen.

## Figures and Tables

**Table 1:** Conservative treatment.

Study	Number of participants	Study type	Therapeutic regimen (all groups)	Group specific therapy	Comparison between groups	Outcomes, conclusions
Schwellnus et al. [10]	43 runners	RCT	Day 0–7: rest ice and medication, daily stretching Days 3, 5, and 7 DTFM	Group 1: placebo 2: anti-inflammatory (Voltaren) 3: anti-inflammatory/analgesic combo (Myprodol)	Group 3 had less pain and increased running time/distance from day 0 to 7	All treatments are effective; analgesic/anti-inflammatory is superior

Gunter and Schwellnus [11]	18 runners	RCT	No running for 14 days after injection and ice for 30 min every 12 hrs.	Group 1: corticosteroid injection (methylprednisolone acetate 40 mg) Group 2: placebo injection	Using a visual analogue scale for pain perception, significant (*P* = 0.01) decrease in pain during running in group 1	Local corticosteroid infiltration effectively decreases pain during running in the first 2 wks of treatment of ITBS Group 1 avg. of 53.6% decreases in pain from day 0 to 14

Pinshaw et al. [12]	24 runners	Case series	Softer running shoes, correct leg-length discrepancies, reduce training distance, ice 30 min. BID		Response to treatment was variable, even some who followed treatment judiciously did not benefit	After 8 weeks, 44% were 100% cured, 22% were 75% cured, and 34% were 50% or less cured

Fredericson et al. [2]	24 (10 M 14 F) injured runners, 30 (16 M 14 F) controls	Case series	Injured runners enrolled in a 6-week rehab to strengthen gluteus medius	Statistically significant (*P* < 0.05) higher hip abductor torque in control group compared to injured runners.	After rehab females increased hip abductor torque 34.9%, males 51.4%	22/24 athletes were pain-free and able to return to running, with recurrence at 6 months
